# A Possible CO_2_ Conducting and Concentrating Mechanism in Plant Stomata SLAC1 Channel

**DOI:** 10.1371/journal.pone.0024264

**Published:** 2011-09-13

**Authors:** Qi-Shi Du, Xina-Wei Fan, Cheng-Hua Wang, Ri-Bo Huang

**Affiliations:** 1 National Engineering Research Center for Non-Food Biorefinery, Guangxi Academy of Sciences, Nanning, China; 2 College of Life Science and Technology, Guangxi University, Nanning, China; 3 Biotechnology and Pharmaceutical Engineering, Nanjing University of Technology, Nanjing, China; 4 Gordon Life Science Institute, San Diego, California, United States of America; University of Melbourne, Australia

## Abstract

**Background:**

The plant SLAC1 is a slow anion channel in the membrane of stomatal guard cells, which controls the turgor pressure in the aperture-defining guard cells, thereby regulating the exchange of water vapour and photosynthetic gases in response to environmental signals such as drought, high levels of carbon dioxide, and bacterial invasion. Recent study demonstrated that bicarbonate is a small-molecule activator of SLAC1. Higher CO_2_ and HCO_3_^–^ concentration activates S-type anion channel currents in wild-type Arabidopsis guard cells. Based on the SLAC1 structure a theoretical model is derived to illustrate the activation of bicarbonate to SLAC1 channel. Meanwhile a possible CO_2_ conducting and concentrating mechanism of the SLAC1 is proposed.

**Methodology:**

The homology structure of *Arabidopsis thaliana* SLAC1 (AtSLAC1) provides the structural basis for study of the conducting and concentrating mechanism of carbon dioxide in SLAC1 channels. The pK_a_ values of ionizable amino acid side chains in AtSLAC1 are calculated using software PROPKA3.0, and the concentration of CO_2_ and anion HCO_3_^–^ are computed based on the chemical equilibrium theory.

**Conclusions:**

The AtSLAC1 is modeled as a five-region channel with different pH values. The top and bottom layers of channel are the alkaline residue-dominated regions, and in the middle of channel there is the acidic region surrounding acidic residues His332. The CO_2_ concentration is enhanced around 10^4^ times by the pH difference between these regions, and CO_2_ is stored in the hydrophobic region, which is a CO_2_ pool. The pH driven CO_2_ conduction from outside to inside balances the back electromotive force and maintain the influx of anions (e.g. Cl^–^ and NO_3_^–^) from inside to outside. SLAC1 may be a pathway providing CO_2_ for photosynthesis in the guard cells.

## Introduction

In biology, a stoma is a tiny pore, found in the epidermal tissues of leaves and stems, which is used for gas exchange. The pore is bordered by a pair of kidney-shaped parenchyma cells known as guard cells, which are responsible for regulating the pore aperture of the opening [1]. Ambient carbon dioxide enters the plant leaves through these stomatal pores, where it is used in photosynthesis. Oxygen produced by photosynthesis in the spongy layer cells (parenchyma cells with pectin) of the leaf interior exits through these same openings. In plant respiration the oxygen enters the plant through the stomata, too. Also, water vapor is released into the atmosphere through these pores in a process called transpiration [2], [3].

The plant SLAC1 is a slow anion channel in the membrane of stomatal guard cell, which controls the turgor pressure in the aperture-defining guard cells of plant stomata [4]–[8], thereby regulating the exchange of water vapour and photosynthetic gases in response to environmental signals such as drought, high levels of carbon dioxide, and bacterial invasion [5], [6]. Studies proved that SLAC1 is activated by phosphorylation from the OST1 kinase [9], [10]. OST1 activity is negatively regulated by the ABI1 phosphatase [11], which is in turn inhibited by the stomatal ABA receptors PYR and RCAR [12] when in the ternary hormone–receptor–phosphatase complex [13], [14]. Thereby, ABA stimulates SLAC1 channel activity. Resulting Cl^–^ efflux through SLAC1 causes membrane depolarization, which activates outward rectifying K^+^ channels, leading to KCl and water efflux to reduce turgor further and cause stomatal closure.

Recent study demonstrated that bicarbonate is a small-molecule activator of SLAC1 [15]–[17]. Elevated intercellular concentration of HCO_3_^–^ with low concentration of CO_2_ and H^+^ activated S-type anion channel, whereas low [HCO_3_^–^] at higher [CO_2_] and [H^+^] did not [15]. Thereby the bicarbonate activates the SLAC1 anion channels. However, the molecular mechanisms that underlie the SLAC1 activation and stomatal CO_2_ signalling have remained relatively obscure. Some logical questions arise from these new findings. How does the concentration of HCO_3_^–^ and CO_2_ activate the SLAC1 to maintain the influx of anions and adjust the pressure in guard cells of stomata? Is there any connection between influx of anions (Cl^–^ and NO_3_^–^) and the concentration of HCO_3_^–^ and CO_2_ in SLAC1 channel?

Recently an atomic-resolution crystal structure of the TehA from *Haemophilus influenzae* at 1.20 Å resolution was solved by Chen *et al*. [18], [19] with the PDB codes 3M71, 3M72, 3M73 and 3M7L (http://www.rcsb.org/pdb/), notably HiTehA (*Haemophilius influenzae* TehA) [19]. Then a homology model of *Arabidopsis thaliana* SLAC1 (AtSLAC1) was developed by Chen *et al.* [18], which is substantially similar to the bacterial homologues. This milestone work provided the structural basis for solving the questions. In this study we perform a theoretical analysis for the activation mechanism of bicarbonate based on the protein structure of AtSLAC1 [18] using physicochemical calculation tools.

## Results

**Figure 1:**
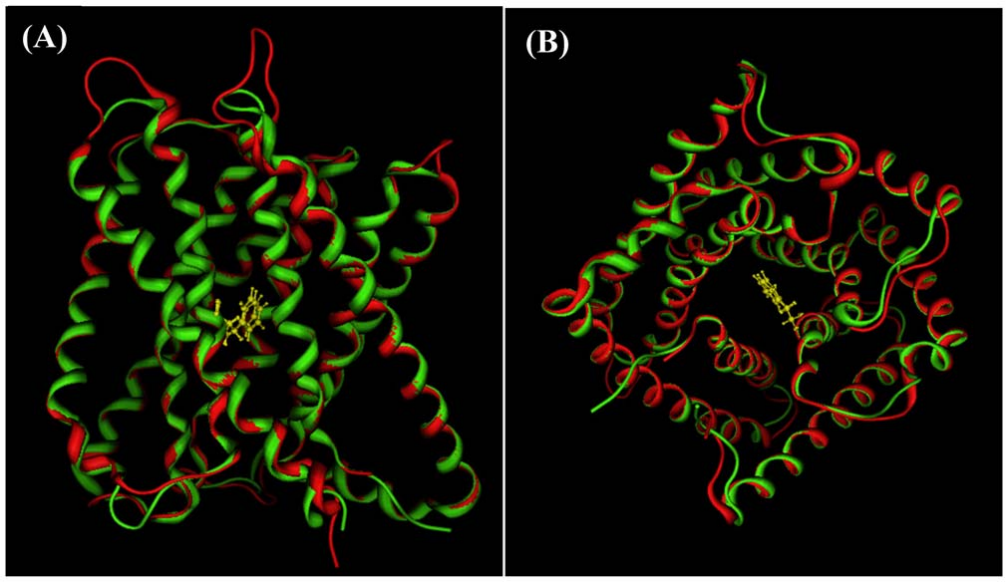
The structural alignment of *Arabidopsis thaliana* SLAC1 (AtSLAC1) homology structure and the template structure *Haemophilius influenzae* TehA (HiTehA). The backbone of AtSLAC1 is shown in red and the HiTehA is in green. (**A**) A side view of AtSLAC1 and HiTehA alignment. The backbones of two structures overlap very nicely. (**B**) A top view of AtSLAC1 and HiTehA alignment. The ten helical hairpins are arranged in two layers with quasi-five-fold symmetry. The residue Phe262 (colored in yellow) is in the center of channel, which is the gate of the channel. The ten helices of the two layers in SLAC1 channel are connected by flexible loops. It is anticipated that the diameter of the SLAC1 channel can be adjusted by pressure change in the guard cell.

The crystal structure of the HiTehA is a trimer consisting of three tightly associated subunits. Each protomer of HiTehA and AtSLAC1 has ten transmembrane helices. The fold of SLAC1 protomer is novel: tandemly repeated helical hairpins are arranged in two layers with quasi-five-fold symmetry. Fig. 1 shows the alignment of AtSLAC1 model structure and its template HiTehA. The backbones of two structures overlap very nicely. The extracellular inter-helix loops are short (1–5 residues), whereas the intracellular inter-helix connections are longer (Fig. 1 A). The top (outside the membrane) and the bottom (inside the membrane) of the SLAC1 channel are filled by water molecules. In Fig. 1 the residue Phe262 (colored in yellow) is in the center of stomatal channel, which is the gate of the channel. The ten helices of the two layers in SLAC1 channel are connected by flexible loops. It is anticipated that the ‘triple-barrel’ structure of the AtSLAC1 channel makes the diameter of the channel is adjusted by pressure change in the guard cells.

The subcellular location of SLAC1 was experimentally determined in the surface of the guard cell using combined SLAC1 protein and green fluorescent protein. Further experiment examined that the SLAC1 is in the plasma membrane [20]. Therefore, the SLAC1 is a plasma-membrane-localized protein in the guard cells, and participates in the control of anion fluxes across the plasma membrane of guard cells [18], [20].

### Amino acid composition of AtSLAC1

**Figure 2:**
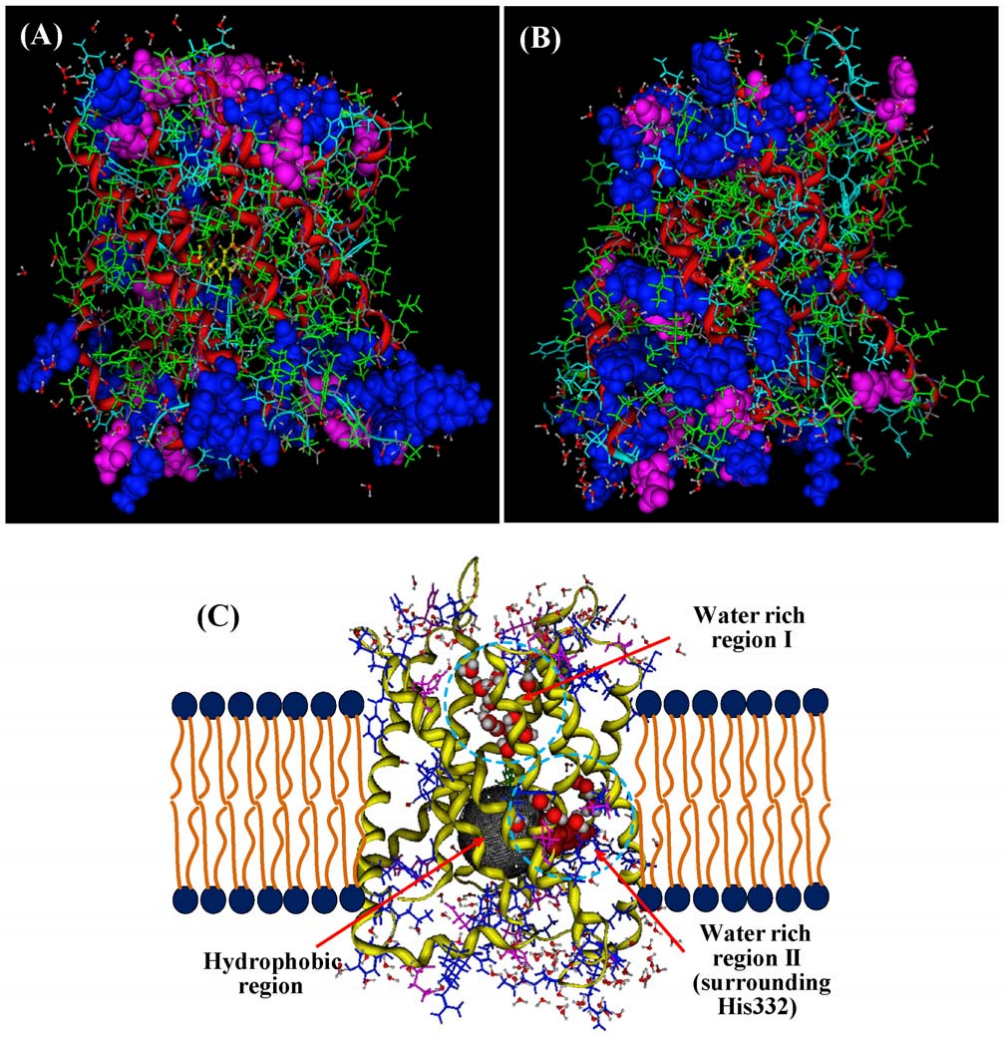
Amino acid distributions in *Arabidopsis thaliana* SLAC1 (AtSLAC1) and in *Haemophilius influenzae* TehA (HiTehA). (**A**) Amino acid distributions of AtSLAC1. The acidic residues are shown in blue space filling render, and the acidic residues in pink space filling render. The polar residues are in light blue line drawing, and the hydrophobic residues are in light green line drawing. (**B**) Amino acid distributions of HiTehA. The acidic residues (pink) and the alkaline residues (blue) are concentrated in top and bottom layers, and the hydrophobic residues (light green) are arranged in the middle of the channel. (**C**) The transmembrane model of AtSLAC1. The top and bottom layers are filled by water molecules. In the channel there are two water-rich regions, in which the water molecules are shown in space filling drawing. The dark gray ball indicates the hydrophobic region, where is an empty cavity.

The amino acid composition and distribution in HiTehA and AtSLAC1 are shown in Fig. 2, where the acidic residues are colored in pink, alkaline residues in blue, polar residues in light blue, and hydrophobic residues in light green. The channel gate 262Phe (in HiTehA) and 462Phe (in AtSLAC1) are shown in yellow. The acidic and alkaline residues are shown in space filling render. Most alkaline residues (blue) and acidic residues (pink) concentrate locate in the top layer and bottom layer of the channel. The hydrophobic residues (light green) are in the middle, the transmembrane part of the channel.

The values of acidic ionizing constant (pK_a_) of residues are essential for the CO_2_ conducting and concentrating. The classification of 20 natural amino acids is listed in Table 1. In Table 1 the pK_a_ values of amino acid side chains are only the model values [21]. The effective pK_a_ values of residues in the protein may be very different from the model values because of the special protein environment. The pK_a_ values of ionizable residues in AtSLAC1 are calculated using software PROPKA3.0 [21]–[23] and listed in Table 2.

**Table 1:** Chemical properties and classification of 20 natural amino acids.

Amino acid	pK_a_ *	Classification
Asp (D)	3.93	Acidic
Glu (E)	4.37	Acidic
His (H)	6.50	Acidic
Arg (R)	12.50	Alkaline
Lys (K)	10.50	Alkaline
Tyr (Y)	10.00	Alkaline
Cys (C)	9.00	Alkaline
Ser (S)	-	Polar
Thr (T)	-	Polar
Asn (N)	-	Polar
Gln (Q)	-	Polar
Trp (W)	-	Polar
Gly (G)	-	Hydrophobic
Ala (A)	-	Hydrophobic
Val (V)	-	Hydrophobic
Leu (L)	-	Hydrophobic
Ile (I)	-	Hydrophobic
Met (M)	-	Hydrophobic
Phe (F)	-	Hydrophobic
Pro (P)	-	Hydrophobic

*The referent pK_a_ values are from references [18]–[20].

**Table 2:** The pKa values of ionizable residues in AtSLAC1.

Acidic Residues			Alkaline Residues		
No.	A.A.	pK_a_	No.	A.A.	pK_a_
351	ASP	3.93	192	CYS	10.68
412	ASP	2.41	196	CYS	12.10
252	GLU	4.37	247	CYS	9.78
257	GLU	5.79	274	CYS	11.49
308	GLU	4.48	298	CYS	9.47
352	GLU	4.66	324	CYS	8.64
380	GLU	4.58	414	CYS	9.61
385	GLU	4.35	418	CYS	10.18
464	GLU	7.03	487	CYS	9.37
219	HIS	6.73	243	TYR	13.83
260	HIS	4.28	250	TYR	10.12
293	HIS	6.02	258	TYR	11.73
332	HIS	3.65	291	TYR	10.79
364	HIS	5.67	304	TYR	11.06
387	HIS	4.52	312	TYR	10.31
496	HIS	5.98	365	TYR	13.99
			373	TYR	10.23
			390	TYR	12.32
			408	TYR	10.38
			426	TYR	10.56
			448	TYR	11.41
			462	TYR	13.40
			469	TYR	10.04
			211	LYS	10.07
			246	LYS	9.86
			255	LYS	10.56
			290	LYS	10.03
			310	LYS	9.20
			320	LYS	9.64
			325	LYS	10.10
			347	LYS	10.05
			355	LYS	9.73
			384	LYS	9.93
			440	LYS	10.44
			461	LYS	9.12
			256	ARG	12.55
			263	ARG	12.14
			289	ARG	12.36
			321	ARG	12.15
			322	ARG	12.57
			375	ARG	11.25
			416	ARG	9.90
			432	ARG	12.41
			472	ARG	12.39

**Figure 3:**
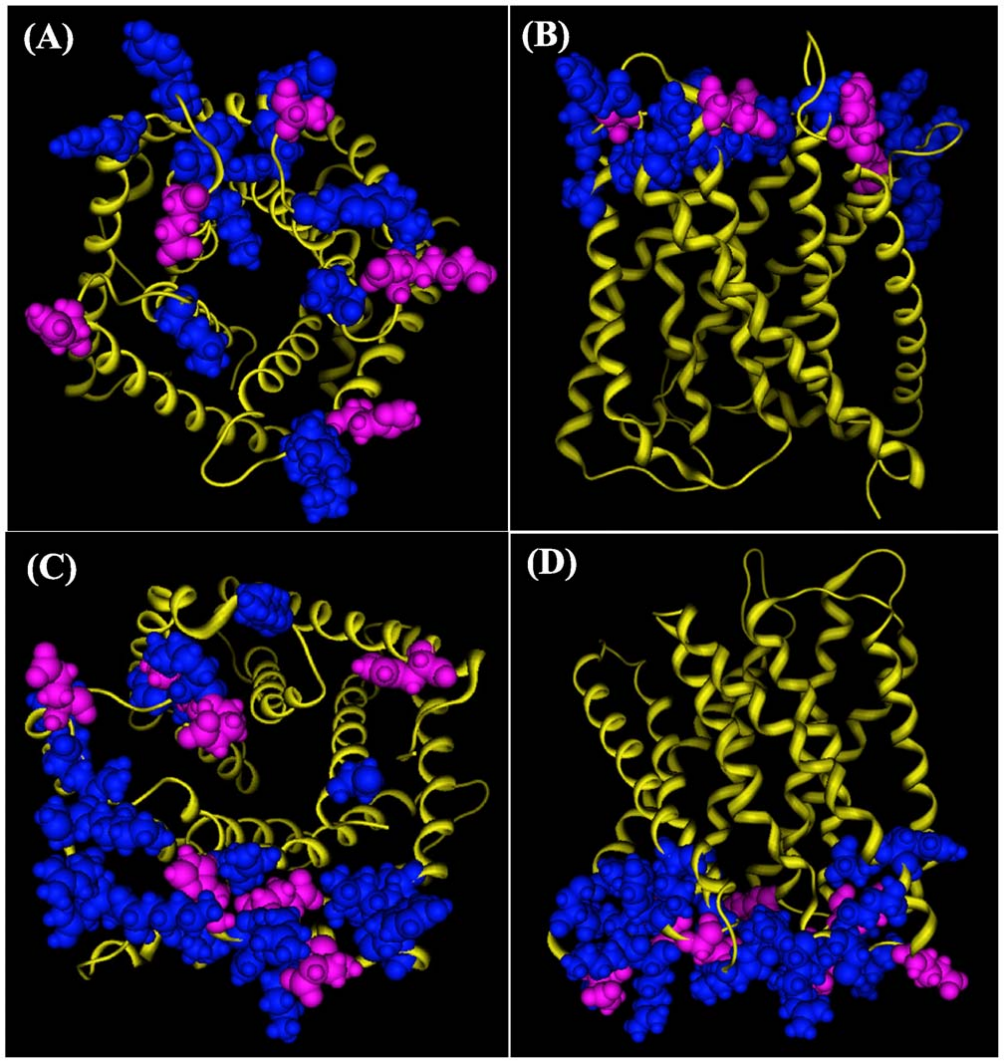
Acidic residues (pink) and alkaline residues (blue) in the top and bottom layers of AtSLAC1 channel. (**A**) A top view of the residue distribution in the top layer. (**B**) A side view of the residue distribution in the top layer. In the top layer there are five acidic residues (His219, His293, Asp351, Asp412, and Glu464) and 14 alkaline residues (Lys211, Arg289, Lys290, Tyr291, Lys347, Lys355, Tyr408, Cys414, Arg416, Cys418, Lys61, Tyr462, Tyr469, and Arg472). **(C**) A bottom view of the residue distribution in the bottom layer. (**D**) A side view of the residue distribution in the bottom layer. In the bottom layer there are 7 acidic residues (Glu252, Glu257, His260, Glu380, Glu385, His387, and His496) and 20 alkaline residues (Cys192, Tyr243, Lys246, Cys247, Tyr250, Lys255, Arg256, Tyr258, Arg263, Lys310, Lys320, Arg321, Arg322, Cys324, Lys325, Tyr373, Arg375, Lys384, Tyr390, and Lys440). Both top and bottom layers are alkaline residue-dominated region, and filled by water molecules.

In Table 2 there are 44 alkaline residues and 16 acidic residues. Most acidic and alkaline residues are located in the top and the bottom of AtSLAC1 channel. The amino acid distribution in the top layer of AtSLAC1 channel is shown in **Fig. 3 A** and **B**. In the top layer there are five acidic residues (His219, His293, Asp351, Asp412, and Glu464) and 14 alkaline residues (Lys211, Arg289, Lys290, Tyr291, Lys347, Lys355, Tyr408, Cys414, Arg416, Cys418, Lys61, Tyr462, Tyr469, and Arg472). In the bottom layer, as shown in **Fig. 3 C and D**, there are 7 acidic residues (Glu252, Glu257, His260, Glu380, Glu385, His387, and His496) and 20 alkaline residues (Cys192, Tyr243, Lys246, Cys247, Tyr250, Lys255, Arg256, Tyr258, Arg263, Lys310, Lys320, Arg321, Arg322, Cys324, Lys325, Tyr373, Arg375, Lys384, Tyr390, and Lys440). In both top layer and bottom layer the alkaline residues exceed the acidic residues much more. The alkaline condition in the top and bottom layers is in favor of CO_2_ absorption and storage.

### CO_2_ conducting mechanism

**Figure 4:**
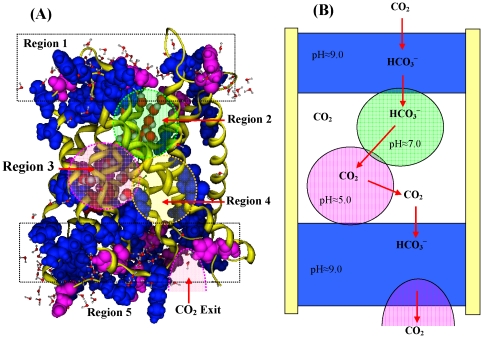
The cartoon model of the AtSLAC1 channel for illustrating CO2 conducting mechanism and concentrating mechanism. (**A**) Five regions of AtSLAC1 channel. The top layer and bottom layer are the region 1 and region 5, respectively. Inside the channel the water-rich region below the top layer is the region 2, and another water rich-region surrounding acidic residue His332 is region 3. The hydrophobic region is the region 4. (**B**) The cartoon model of plant AtSLAC1 channel. The region 1 and region 5 (top and bottom layer) are modeled as alkaline solution (pH≈9.0). The region 2 is neural aqueous solution (pH≈7.0), and region 3 is an acidic region (pH≈5.0), because the residue His332 possesses the very lower pK_a_ value (pK_a_ = 3.65). The hydrophobic region 4 is a CO_2_ storage pool.

Fig. 4 shows the cartoon model of the AtSLAC1 stomatal channel, which is used to illustrated the conducting mechanism of carbon dioxide. The SLAC1 channel is divided into five regions. The first region is the top layer of SLAC1 channel, which is modeled as an alkaline aqueous solution, because it is dominated by alkaline residues and filled by water molecules. Below the first region there is a water pool, the second region in the channel, which is surrounded by polar residues and filled by water molecules. In Fig. 4 the third region is also filled by water molecules, surrounding the acidic residue His332 (space filling render in dark red), which has the second lowest pK_a_ value (pK_a_ = 3.65) in Table 2. The fourth region is a hydrophobic region, formed mainly by hydrophobic residues, which is an empty cavity. The fifth region is the bottom layer of the channel, which is formed by alkaline and acidic residues and filled by water molecules. The fifth region is an alkaline solution, because where are much more alkaline residues (blue) than the acidic residues (pink). However, in the bottom layer there are one or two acidic exits of CO_2_ formed by the acidic residues.

The carbon dioxide conducting mechanism can be illustrated based on the cartoon model of SLAC1 channel in Fig. 4. The CO_2_ conductance is a six-step procedure. The CO_2_ is first absorbed from the atmosphere into the alkaline solution in the region 1, forming hydrogen carbonate ion HCO_3_^−^. In the second step the HCO_3_^−^ migrates to the aqueous solution in the region 2. Then in the third step the ion HCO_3_^−^ enters the acidic region 3 centered by His332 (pK_a_ = 3.65), where it dissociates to CO_2_ in the acidic condition. In the fourth step the saturated CO_2_ in the acidic region 3 comes to the hydrophobic region 4, which is a carbon dioxide storage pool. In the fifth step, from the carbon dioxide pool the CO_2_ dissolves in the alkaline solution in the region 5, forming hydrogen carbonate ion HCO_3_^−^. Finally in the sixth step, the ion HCO_3_^−^ dissociates to CO_2_ in the acidic exit of the region 5, and comes to the cell plasma through the acidic exit. The step 1, transfer of CO_2_ from atmosphere to region 1, is a gas-solution equilibrium process. The step 2 of HCO_3_^−^ migration from region 1 to region 2 is caused by concentration gradient. The steps 4 and 5 are also the gas-solution equilibrium process. The reversible conversion of CO_2_ to HCO_3_^−^ is driven by pH differences between different regions. It is much faster than the conversion in uniform solution with constant pH value.

### CO_2_ concentrating mechanism

The CO_2_ conductance from atmosphere to cell plasma through SLAC1 channel enhances the CO_2_ concentration remarkably. Assuming in atmosphere the concentration of carbon dioxide is [CO_2_]_air_ and the pH value in alkaline solution of the region 1 is pH = 9.0, the concentration of hydrogen carbonate ion HCO_3_^−^ is calculated as follows.
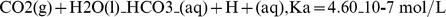


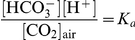


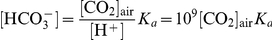



In the acidic region 3 surrounding His332, the hydrogen carbonate ion HCO_3_^−^ dissociates to CO_2_. This is just the reverse reaction of the above equation, *K_b_* = *K_a_* ^−1^. Assuming in the acidic region 3 the pH value is 5.0, the concentration [CO_2_]_3_ in region 3 is calculated as follows.


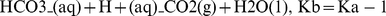



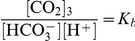



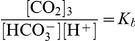



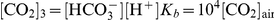


Therefore, the CO_2_ concentration in the region 3 of SLAC1 channel is 10^4^ times higher than the concentration in the atmosphere.

The above calculation for CO_2_ concentrating mechanism is not rigorous because of the following two problems. The first problem is that the calculation uses the assumed pH values in the alkaline solutions (pH = 9) and in the acidic regions (pH = 5). The second problem is that the chemical equilibrium equation holds for macro system, however, the SLAC1 channel is not a macro system. Although the above calculation is not rigorous, it still can be used to illustrate the CO_2_ concentrating in the SLAC1 channel qualitatively. If the alkaline residues and acidic residues in the first, third, and fifth regions are treated as the donors and receptors of H^+^, OH^−^, and HCO_3_^−^, we can get the same qualitative results.

If the concentration (partial pressure) of CO_2_ in atmosphere is [CO_2_]_air_ = 0.0003 atm, according to the above calculations, the CO_2_ concentration in the hydrophobic region 4 (CO_2_ pool) is
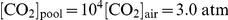



The very high concentration of CO_2_ in AtSLAC1 channel may be over estimated. However, the carbon dioxide concentration in plant SLAC1 channel must be higher than that in the atmosphere.

### Activation mechanism of SLAC1

Recent study revealed that the bicarbonate is a small-molecule activator of SLAC1 [15]. The activation mechanism of HCO_3_^–^ to AtSLAC1 can be illustrated based on the CO_2_ conduction and concentration model of AtSLAC1 proposed in this study. The *Arabidopsis thaliana* SLAC1 was identified as a slow anion channel [18]. Study shows that electrostatic features of the pore coupled with electrophysiological characteristics indicate that selectivity among different anions is largely a function of the energetic cost of ion dehydration [18]. The relative anion permeability sequence of SLAC1 is I^–^>NO_3_^–^>Br^–^>Cl^–^ [18], [24], [25]. The SLAC1 channel transports anions (Cl^–^ and NO_3_^–^) from inside of guard cell to outside cross the membrane. The one-way conduct will make the electrostatic potential inside the guard cell is higher than the outside. The back electromotive force will stop the conduction. On the other hand, the conduction of anion HCO_3_^–^ through SLAC1 channel is from outside to inside of the guard cell driven by pH difference. The difference between anion HCO_3_^–^ and other anions (I^–^, NO_3_^–^, Br^–^, and Cl^–^) is that the anion HCO_3_^–^ is pH sensitive, which has higher concentration in alkaline solution, and dissociates to CO_2_ in acidic solution. Therefore, pH value has strong modulation ability to anion HCO_3_^–^ than to other anions. The conduction of CO_2_ (in HCO_3_^–^ form) in SLAC1 channel from outside to inside is a necessary condition to balance the back electromotive force and maintain the influx of other anions (Cl^–^, NO_3_^–^, I^–^ and Br^–^) from inside to outside. In this way the bicarbonate plays the role of activator for SLAC1 channel.

## Discussion

Usually the reversible conversion of between CO_2_ and HCO_3_^–^ is a very slow process without the catalysis by carbonic anhydrases. This is the phenomena of the conversion between CO_2_ and HCO_3_^–^ in a uniform solution with constant pH value. The proposed model of SLAC1 channel consists of several regions with different pH values. This is only possible in a micro channel. Just the different pH values elevate the concentration of CO_2_, and make the conversion between CO_2_ and HCO_3_^–^ much faster than in uniform macro solution. This is like the case when a drop of hydrochloric acid is put in NaHCO_3_ solution, the CO_2_ escapes out quickly.

The function of CO_2_ conduction and concentration of SLAC1 channel is highly interesting, because it implies a possible pathway of CO_2_ supply in plant. As we known the stomatal aperture is the regular pathway of CO_2_ supplying to cells in leaves for photosyntheric reactions. However, the pathway of CO_2_ influx to the guard cells self is unclear. The proposed mechanism of CO_2_ conduction and concentration indicates that the SLAC1 channel may be a possible pathway providing CO_2_ for photosynthesis in guard cells. The high concentration of CO_2_ (or HCO_3_^–^) in the plant SLAC1 channel not necessarily means the high concentration of CO_2_ (or HCO_3_^–^) in guard cells, because the transfer of CO_2_ (or HCO_3_^–^) in cell plasma of guard cells to the enzyme RuBisCo needs the help of enzyme CA (carbonic anhydrase) [26]–[31]. However, the CO_2_ concentrating in SLAC1 channel may be a mechanism dealing with the instant fluctuation of carbon dioxide in environment.

The possible function of CO_2_ conduction and concentration in SLAC1 channel is supported by the water-channel protein aquaporin [32]–[35]. The role of aquaporin in CO_2_ diffusion in higher plants was first examined by Terashima and Ono [36]. A significant decrease of *g_i_* (internal CO_2_ conductance) was detected in the presence of HgCl_2_, an inhibitor of most aquaporins, which is the evidence indicating involvement of aquaporins in CO_2_ diffusion across the plasma membrane [36]. Then the role of aquaporin in CO_2_ diffusion inside plant leaves was further confirmed by Hanba et al. [37].

In the cartoon model of AtSLAC1 channel (Fig. 4), the top region and bottom region are modeled as the alkaline solutions. However, the two regions are best to be described as the alkaline buffer solutions, because of the alternately distribution of alkaline residues and acidic residues. The alkaline residue-dominated buffer solution not only can maintain the constantly higher pH value, but also can accommodate more CO_2_ (or HCO_3_^–^). In the AtSLAC1 model the acidic His332 in the region 3 plays an important role, by which the CO_2_ concentration in the hydrophobic region (CO_2_ pool) is enhanced greatly. Actually, histidine can play the role of both proton donor and acceptor. The transfer of HCO_3_^–^ to CO_2_ at the His332 may be the speed-control step in the slow anion channel.

Carbon dioxide is a key reactant in plant photosynthesis. The continuing rise in of green house gas CO_2_ in atmosphere is predicted to have diverse and dramatic effects on the productivity of agriculture, plant ecosystems, and global climate [38]–[40]. The CO_2_ conducting mechanism and concentrating mechanism in plant SLAC1 channel, derived in this study based on the structure of AtSLAC1, may provide useful insight into this important research topic.

## Materials and Methods

### Amino acid classification and pK_a_ calculation

The plant SLAC1 anion channel has a novel amino acid composition, and its unique mechanism for CO_2_ conductance can be illustrated using the physicochemical properties of amino acids. The properties of 20 natural amino acids and the pK_a_ values of side chains are listed in Table 1. The 20 amino acids are classified into four types: acidic, alkaline, polar, and hydrophobic. In this study the acidic residues includes Asp, Glu and His, and the alkaline residues are Arg, Lys, Tyr, and Cys [21]–[23]. Five amino acids (Ser, Thr, Asn, Gln, and Trp) are classified as the polar residues. The remaining 8 amino acids are hydrophobic residues. In Table 1 the pK_a_ values of ionizable amino acid side chains are model values [23], which may be very different from the effective pK_a_ values in protein environment.

The effective pK_a_ values of ionizable amino acids in HiTehA and AtSLAC1 are calculated using software PROPKA3.0 [21]-[23]. 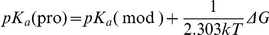



In the above calculation equation the ΔG is the free energy change of a residue side chain from exposed environment to the protein fold environment. When a protein folds, the titratable amino acids in the protein are transferred from a solution-like environment to an environment determined by the 3-D structure of the protein. In the unfolded protein the titratable side chain of amino acid typically exposes to water. When the protein folds the side chain could be buried deeply in the protein interior with no exposure to solvent. Furthermore, in the folded protein the side chain may be closer to other titratable groups in the protein and will also interact with permanent charges (e.g. ions) and dipoles in the protein. All of these effects alter the pK_a_ value of the amino acid side chain. The pK_a_ calculation methods generally calculate the effect of the protein environment on the model pK_a_ value of an amino acid side chain.

### Calculation of pH value in CO_2_ solution

The CO_2_ conducting and concentrating mechanism of plant SLAC1 channel relate with the unique physicochemical properties of carbon dioxide. When carbon dioxide dissolves in water, it exists in equilibrium with carbonic acid.
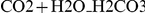



Carbonic acid is diprotic having two protons, which may dissociate from the parent molecule. Thus there are two dissociation constants. 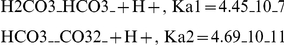



The first one is the dissociation into the hydrogen carbonate ion HCO_3_^−^, and the second is the dissociation of the bicarbonate ion into the carbonate ion CO_3_^2−^. However, in aqueous solution carbonic acid only exists in equilibrium with carbon dioxide, and the concentration of H_2_CO_3_ is much lower than the dissolved CO_2_ concentration. Since it is not possible to distinguish between H_2_CO_3_ and dissolved CO_2_ by conventional methods, the dissolving and ionizing equation of CO_2_ in aqueous solution may be rewritten as follows, 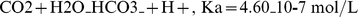



Whereas this K_a_ is quoted as the dissociation constant of carbonic acid, and it might better be referred to as the acidity constant of dissolved carbon dioxide, as it is particularly useful for calculating the pH of CO_2_-containing solutions. In the alkaline solution, the higher pH value (lower concentration [H^+^]) is favor in CO_2_ dissolution (higher concentration [HCO_3_^−^]). On the other hand, lower pH value (higher concentration [H^+^]) makes the HCO_3_^−^ to dissociate to CO_2_.
